# The application of the single branch-first combined with the mid-arch clamping technique and the embedded anastomosis technique for DeBakey type II aortic dissection

**DOI:** 10.1186/s13019-020-1082-9

**Published:** 2020-02-22

**Authors:** Quan Li, Hong Qu, Tianqi Liu, Min Li, Shanliang Chen, Peijie Li, Li Xu, Hengbao Wang

**Affiliations:** Division of Cardiac Surgery, Shandong Provincial Qianfoshan Hospital, the First Hospital Affiliated with Shandong First Medical University, Affiliated Hospital of Shandong University, Jinan, Shandong, China, 16766 Jingshi Road, Jinan, 250014 Shandong China

**Keywords:** DeBakey type II aortic dissection, Single branch-first, Mid-arch clamping, Embedded anastomosis

## Abstract

**Background:**

Patients with DeBakey type II aortic dissection or ascending aortic aneurysms involving the right innominate artery require hemiarch replacement and placement of a right innominate artery graft. Traditional aortic hemiarch replacement surgery must be performed under right axillary artery cannulation perfusion and moderate or deep hypothermia circulatory arrest. However, the axillary artery perfusion is always associated with left subclavian artery “steal blood”, and it cannot guarantee blood supply to the left cerebral hemisphere in patients with an incomplete circle of Willis, and hypothermia and hypoperfusion cause damage to the brain and spinal cord; therefore, postoperative complications of the nervous system are common. Herein, we present a hemiarch replacement procedure with the use of the single branch-first combined with the mid-arch clamping technique. This procedure can not only reduce the axillary artery incision but also eliminate the need for mid-deep hypothermia and circulatory arrest.

**Case presentation:**

A 41-year-old male patient underwent surgery with this technique. Computed tomography angiography performed upon admission showed calcified plaques scattered throughout the aorta and showed DeBakey type II aortic dissection involving the right innominate artery, accompanied by cardiac tamponade. The patient underwent aortic root repair, ascending aorta replacement, and hemiarch replacement as well as the placement of a right innominate artery graft. Aortic root anastomosis was performed with the embedded anastomosis technique. There were no postoperative complications. The patient was discharged 11 days after the operation. During more than 3 months of follow-up, there were no cases of aortic valve regurgitation or anastomotic fistula.

**Conclusions:**

The single branch-first combined with the mid-arch clamping technique for the right innominate artery can reduce the axillary artery incision and avoid damage to the body under mid-deep hypothermia and circulatory arrest. The embedded anastomosis technique is easy to perform, results in a limited amount of bleeding and requires almost no extra needling. We believe that these techniques can serve as good alternative strategies for patients with DeBakey type II aortic dissection or ascending aortic aneurysms involving the right innominate artery.

## Background

Currently, the best way to address DeBakey type II aortic dissection or ascending aortic aneurysms involving the right innominate artery is via hemiarch replacement and the placement of a right innominate artery graft. Because the right innominate artery is involved, this artery is not typically selected for cannulation and perfusion. The traditional approach is to perform the operation under conditions of cerebral perfusion with right axillary artery cannulation and circulatory arrest with mid-deep hypothermia. However, the axillary artery perfusion cannot guarantee blood supply to the left cerebral hemisphere in patients with an incomplete circle of Willis, and hypothermia and hypoperfusion cause damage to the brain and spinal cord; therefore, postoperative complications of the nervous system are common [1, 2]. Herein, we present a hemiarch replacement procedure with the use of the single branch-first combined with the mid-arch clamping technique for the right innominate artery; this procedure can not only reduce the incision of the axillary artery but also eliminate the need for mid-deep hypothermia and circulatory arrest. The traditional anastomosis method performed between the aortic root and artificial vessel has been the “sandwich” method; the resulting anastomosis is firm and does not easily bleed. However, the inner and outer linings of the aortic wall need to be padded with felt pieces, which is difficult to perform, and the sutures are not easy to tighten. Due to the shielding of the felt pieces, the location of bleeding is not easy to identify. Thus, we applied the embedded anastomosis technique, which is highly convenient to perform, results in a limited amount of bleeding and requires almost no extra needling.

## Case presentation

**Fig. 1:**
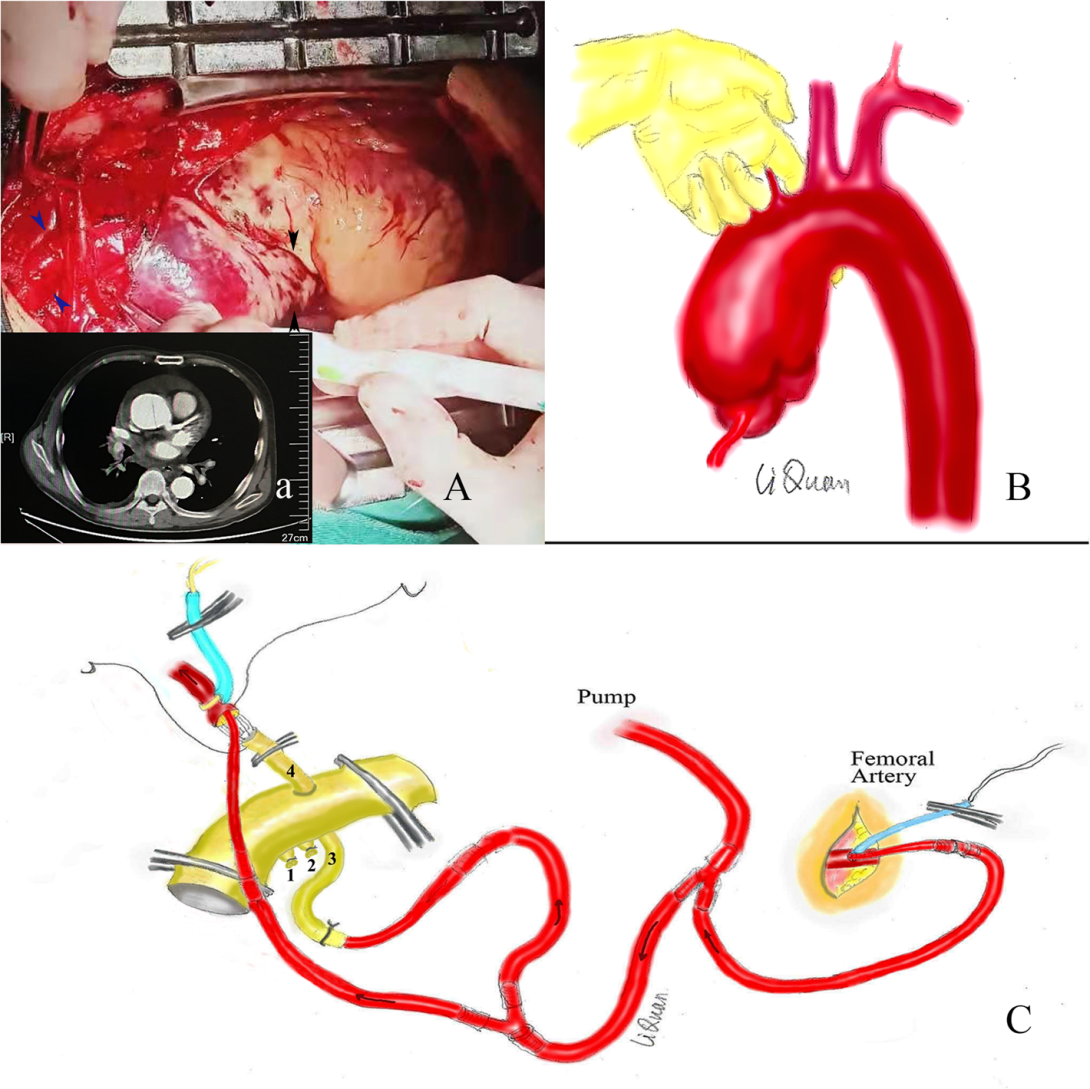
(a) Results of CTA (black arrows indicate cross-sectioning). (A) Image showing the open pericardial exploration. Aortic root of the ascending aorta (black arrow); Right innominate artery (blue arrow). (**b**) Method of separation of the aortic arch and tracheal space. (**c**) Method of cannulation for extracorporeal circulation and perfusion and the single branch-first technique

A 41-year-old male patient was transferred from the local hospital to our hospital due to “sudden chest and back pain for 5 hours”. The patient had a history of hypertension for 10 years, and the highest reported blood pressure was 170/100 mmHg. He was treated with nifedipine and carvedilol for a long period of time, and his blood pressure was not well controlled. He also had a history of cerebral infarction for 6 years that resulted in paralysis of the left limbs. The patient had renal dysfunction for more than 7 months and had undergone regular dialysis treatment. An aortic computed tomography angiography (CTA) examination upon admission showed calcified plaques scattered throughout the aorta and DeBakey type II dissection of the ascending aorta involving the right innominate artery, accompanied by cardiac tamponade (Fig. 1a). Cardiac ultrasound showed pericardial tamponade, ascending aorta dilation, a floating intima 2 cm above the junction of the sinus tube, and no aortic valve regurgitation. The patient underwent emergency aortic root repair, ascending aorta replacement, and hemiarch replacement as well as the placement of a right innominate artery graft.

The right femoral artery was separated via a right inguinal incision. An anterior medial incision was created, and the right innominate artery was separated. The space between the arch and trachea was separated between the right innominate artery and the left common carotid artery with the left index finger as the middle arch blocking position, as was performed previously (Fig. 1b) [3]. Open pericardial exploration showed the following: haematic pericardial effusion of approximately 300 ml and haematoma of the entire ascending aorta involving the right innominate artery. In fact, the haematoma was confined to the innominate artery (Fig. 1A). After heparinization, an 18 Fr arterial cannula was inserted into the femoral artery, another 18 Fr cannula was inserted into the third branch of the #26 artificial four branch vessels (VASCUTEK Ltd., Inchinnan, Scotland), and another 12 Fr arterial cannula was used for standby (Fig. 1c) [3]. The four branch vessels were prefilled with CO_2_, and the gas in the duct (including the four branch vessels) was removed via the return of blood from the femoral artery cannula.

**Fig. 2:**
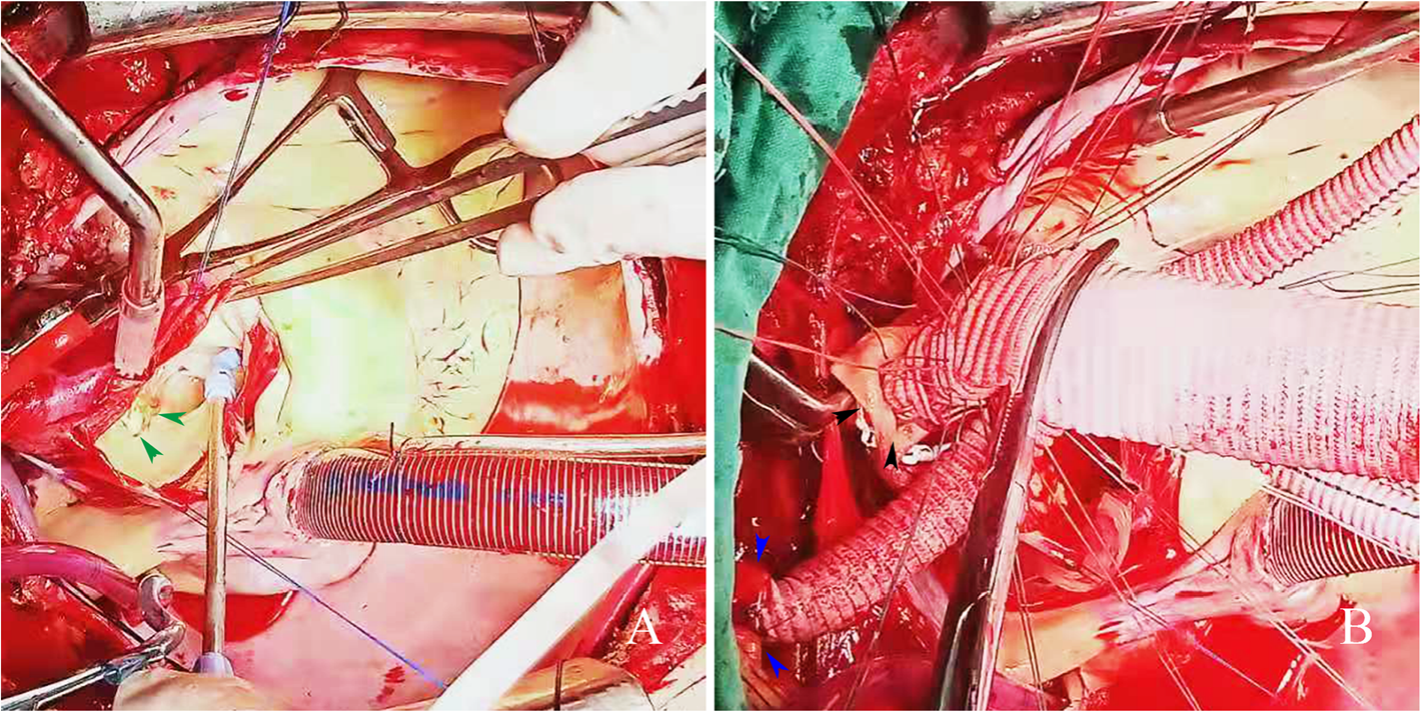
(**a**) Crevasse position (green arrow). (**b**) Intermittent anastomosis of the proximal end of the arch. Right innominate artery (blue arrow); Opened stump of the right innominate artery (black arrow)

First, the “single branch-first” technique for the right innominate artery was used. The artery was transected 1 cm from the root of the right innominate artery, and a 12 Fr arterial cannula was inserted into the distal true lumen (return of blood from the femoral artery cannula was used to restore blood perfusion). Continuous suturing with 5–0 Prolene suture was performed to anastomose the fourth branch of the artificial vessel and the distal end of the right innominate artery (Fig. 1c). After anastomosis, the third branch of the artificial vessel was used for innominate artery perfusion. A two-stage venous cannula was inserted into the right atrium, cardiopulmonary bypass began, and the temperature decreased. A left cardiac drainage tube was placed in the upper right pulmonary vein for decompression. When the core temperature was 34 °C (nasopharyngeal temperature was approximately 32 °C), the “mid-arch clamping” technique was initiated. The aortic arch was blocked between the right innominate artery and the left common carotid artery. The longitudinal incision of the ascending aorta was made superior and inferior to the right innominate artery stump to a location 2 cm above the noncoronary sinus. Ascending aortic dissection involving the right innominate artery was discovered, and the dissection was confined to the innominate artery. The crevasse was located approximately 2 cm above the junction of the sinus tube in the noncoronary sinus, with a length of approximately 2 cm, and was surrounded by a calcified ulcer (Fig. 2a). The dissection involved the junction of the sinus tube in the left coronary sinus, the noncoronary sinus and the commissure of the right noncoronary sinus. A three-leaf aortic valve, without prolapse, and a valve ring without dilation were found. A thrombus of approximately 10 g was removed from the sandwich. The aortic root was poured into the myocardial protective fluid. Then, the dissected intima was excised proximal to 1 cm above the junction of the sinus tube and distal to the near-end of the arch.

Then, anastomosis of the proximal end of the arch was performed. The distal end of the artificial vessel was blocked close to the third and fourth branches and was trimmed to retain a suture margin of 2 cm. The arch cavity was lined with a free artificial vessel ring that was approximately 1 cm long. A 2–0 ETHIBOND EXCELTM (v-5) suture was used to anastomose the distal end of the artificial four branch vessels with the proximal end of the arch with the use of the intermittent stitch method (Fig. 2b). Afterwards, the artificial vessel and the mid-arch clamping forceps were opened, and the anastomosis was checked for bleeding. Intermittent reinforcement with 3 stitches was performed to stop the bleeding.

**Fig. 3:**
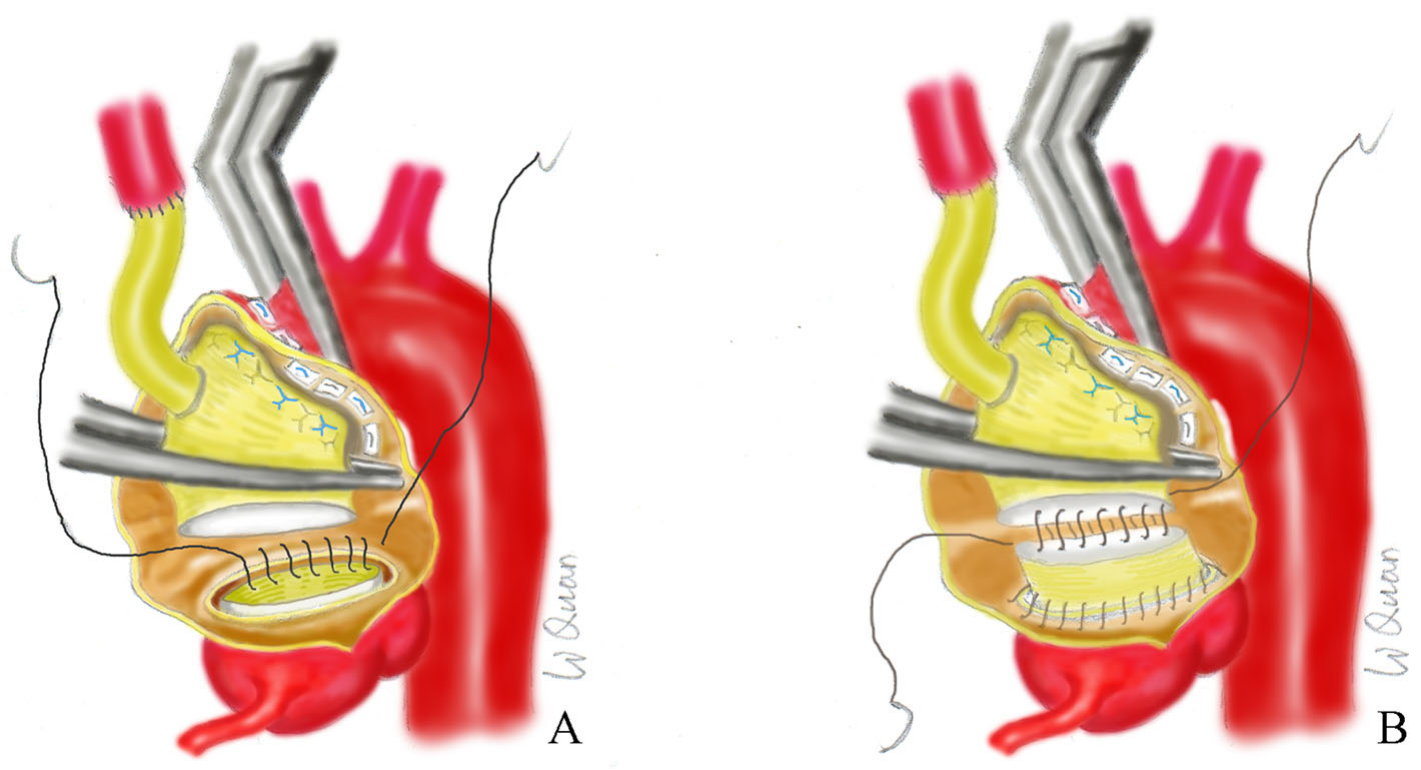
(**a**) Step 1 of the embedded anastomosis technique. (**b**) Step 2 of the embedded anastomosis technique

Next, anastomosis of the aortic root was performed. The commissure of the right noncoronary sinus was suspended by a 4–0 Prolene suture with a gasket. Another 3 sutures were placed to clip the dissection of the noncoronary sinus. Next, we applied the “embedded anastomosis” technique. A segment of free artificial blood vessel approximately 5 cm in length was taken, and the lumen was reversed and inserted into the left ventricle through the aortic valve ring. The proximal end of the artificial vessel was located 1 cm above the junction of the sinus tube, close to the intima of the aortic cavity. Continuous stitching was performed with 4–0 Prolene HS suture (Fig. 3a). After tying a knot, the artificial vessel was turned out and trimmed to an appropriate length. The proximal end of the four branch vessels was blocked close to the third and fourth branches, and the residual end was trimmed to the suture margin of 1 cm. A 4–0 Prolene HS suture was used for continuous suturing (Fig. 3b). After deairing, the blocking forceps of the four branch vessel was opened, and the heart began to beat again. Each cannula was removed after protamine and heparin neutralization, and each incision was closed.

The patient experienced a smooth operation. The total cardiopulmonary bypass time was 180 min, and the aortic clamping time was 122 min. The drainage volume 8 h after the operation was 200 ml. There were no postoperative complications. The patient was discharged 11 days after the operation. During more than 3 months of follow-up, there were no cases of aortic regurgitation or anastomotic fistula.

## Discussion

The common methods used for the cannulation of arterials in cardiac surgery include ascending aorta cannulation, axillary artery cannulation, innominate artery cannulation and femoral artery cannulation. Axillary artery cannulation is the most commonly used method in traditional hemiarch replacement surgery. The advantages of axillary artery cannulation are as follows. 1. After the circulation is stopped at deep hypothermia, low-flow perfusion of the brain can be achieved through axillary artery cannulation to ensure the continuous supply of oxygen to the brain. 2. There is universally no dissection of the axillary artery, so there is no risk of insertion of a false lumen. However, the biggest limitations of axillary artery cannulation are as follows. 1. Perfusion of the left side of the brain requires a complete circle of Willis, and the circle of Willis is incomplete in approximately 15% of patients, so axillary artery perfusion cannot guarantee oxygen supply to the left side of the brain in this subset of patients. Therefore, axillary artery perfusion is relatively safe in deep hypothermia surgery, but it is not suitable for mild hypothermia surgery. 2. There is a risk of “steal blood” flowing through the left vertebral artery to the left upper limb. 3. Axillary artery cannulation alone cannot solve the problem of ischaemia of the spinal cord and abdominal organs when circulation is stopped. Therefore, hemiarch surgery with axillary artery cannulation alone requires substantial experience by the operator, who is required to complete the anastomosis of the arch and restore the blood supply of the lower body as soon as possible. Because the ascending aorta needs to be replaced simultaneously during hemiarch surgery, cannulation of the ascending aorta is not an option. An innominate artery cannula can be used for hemiarch replacement surgery but should not be used if the dissection involves the innominate artery because of the risk of inserting a false lumen. However, femoral artery cannulation combined with the mid-arch clamping technique can ensure continuous blood supply to the spinal cord and abdominal organs without the need for deep hypothermia and circulatory arrest.

The advantages of the “single branch-first” technique are as follows. 1. Because of the deep location and the separation difficulty of the axillary artery as well as the risk of brachial plexus nerve injury, it is beneficial to avoid axillary artery incision. In the single branch-first technique, incision of the axillary artery is avoided, which can simplify the operation and reduce surgical trauma and the operation time. 2. The anastomosis of the right innominate artery is completed before cardiopulmonary bypass, so this technique reduces the time of cardiopulmonary bypass. 3. After transecting the root of the innominate artery, the perfusion cannula can be directly inserted into the true lumen, so the risk of inserting the cannula into the false lumen can be avoided.

There have been very few reports on the application of the “mid-arch clamping” technique in arch surgery [3–5]. The advantages of this technique are as follows. 1. The brain, spinal cord and abdominal organs are continuously perfused under the condition of mild hypothermia, which reduces the complications associated with the nervous system and abdominal organs. 2. The time for cooling and rewarming is reduced, and the time of extracorporeal circulation is reduced. 3. The position of the arch between the right innominate artery and the left common carotid artery is relatively shallow, and there is no laryngeal return or vagus nerve passage behind the arch. Moreover, the gap between the trachea and the mid-arch is very loose, and blunt separation is very easy.

The disadvantages of the “branch-first combined with the mid-arch clamping” technique are as follows. 1. The surgical procedure is relatively complex, and the surgical field is crowded. 2. After mid-arch clamping, the condition in the distal end of the arch cavity cannot be detected; thus, we can only rely on preoperative CTA imaging results. 3. Compared to open anastomosis, close anastomosis of the mid-arch is difficult. To solve this problem, we gave up a continuous suturing technique and used an intermittent stitch method with 2–0 ETHIBOND EXCELTM (v-5) suture, which was similar to the suturing method used for aortic valve replacement and alleviated the difficulty of continuous suturing to some extent. Although the intermittent suturing technique prolonged the anastomosis time, this method made the suturing of each stitch more flexible, and each needle was firm, not loose, and resulted in a limited amount of bleeding after knot placement.

The “embedded anastomosis” technique (Fig. 3a, b) used for the artificial blood vessel was applied to the aortic root to make suturing easier, more even and tighter. This method has often been used by us for total arch replacement with very good results, limited bleeding and almost no extra needling requirements. At the same time, the disadvantage is that there is an additional anastomosis compared with the artificial vessel from the arch that is directly anastomosed with the aortic root.

No procedure is absolutely perfect, and each has its own advantages, disadvantages and application scores. Therefore, it is very beneficial to have additional surgical methods as supplements for traditional methods. For patients with DeBakey type II aortic dissection or ascending aortic aneurysms involving the right innominate artery, we believe that the “single branch-first combined with the mid-arch clamping” technique is a very good choice for clinical doctors.

## Conclusions

The single branch-first combined with the mid-arch clamping technique for the right innominate artery can reduce the axillary artery incision and avoid damage to the body under mid-deep hypothermia and circulatory arrest. The embedded anastomosis technique is easy to perform, results in a limited amount of bleeding and requires almost no extra needling. We believe that these techniques can serve as good alternative strategies for patients with DeBakey type II aortic dissection or ascending aortic aneurysms involving the right innominate artery.

## Data Availability

Not applicable.

## Abbreviations

CTA: Computed tomography angiography
